# Impact of biofilm formation in fungal corneal ulcers on treatment outcomes: a systematic review and meta-analysis

**DOI:** 10.1099/jmm.0.002106

**Published:** 2025-12-18

**Authors:** Anna Nur Utami, Alya Nabilah Tasya, Rina La Distia Nora, Tri Wibawa

**Affiliations:** 1 Doctoral Program in Medical and Health Sciences, Faculty of Medicine, Public Health and Nursing, Universitas Gadjah Mada, Yogyakarta, Indonesia; 2 JEC Eye Hospitals and Clinics, Jakarta, Indonesia; 3 Faculty of Medicine, Public Health and Nursing, Universitas Gadjah Mada, Yogyakarta, Indonesia; 4 Department of Ophthalmology, University of Indonesia, Jakarta, Indonesia; 5 Department of Microbiology, Faculty of Medicine, Public Health and Nursing, Universitas Gadjah Mada, Yogyakarta, Indonesia

**Keywords:** antifungal resistance, biofilm, corneal ulcer, fungal, treatment outcome

## Abstract

**Introduction.** Fungal keratitis, particularly in tropical and subtropical regions, poses significant therapeutic challenges due to biofilm formation by fungal pathogens. These biofilms confer increased resistance to antifungal treatments and are associated with poorer clinical outcomes.

**Hypothesis/Gap Statement**. Despite growing recognition of their impact, there remains a lack of comprehensive synthesis on the role of fungal biofilms in corneal ulcers.

**Aim.** This study aims to determine the impact of and how biofilm formation influences the chronicity and treatment outcomes in fungal corneal ulcers.

**Methodology.** A comprehensive literature search was performed across PubMed, ScienceDirect, Scopus and the Cochrane Library in April 2025. Only English articles were included, and animal studies were excluded. Eligible studies included clinical and *in vitro* investigations that assessed biofilm formation in fungal corneal ulcers and its impact on antifungal susceptibility and treatment outcomes. This systematic review and meta-analysis were conducted in accordance with PRISMA (Preferred Reporting Items for Systematic reviews and Meta-Analyses) 2020 guidelines and registered under PROSPERO (an international systematic review registry, ID:CRD420251017502). Independent data extraction was done by two reviewers. Data on MICs were synthesized using random-effects models, and heterogeneity was assessed with I² statistics and Cochran’s Q test. Clinical outcomes were analysed narratively due to reporting variability.

**Results.** Seven studies were included, spanning Brazil, India, China and Mexico, and covering both *in vitro* and clinical designs. Meta-analysis showed significantly increased MIC values for biofilm-forming fungal isolates: amphotericin B [pooled log_₂_ fold change=5.31; 95% confidence interval (CI): 2.92–7.70], voriconazole (6.06; 95% CI: 2.25–9.87) and natamycin (1.25; 95% CI: 0.48–2.02). High heterogeneity was noted for amphotericin B and voriconazole, while results for natamycin were consistent. Narrative synthesis of clinical data indicated that biofilm formation is associated with prolonged healing times, increased recurrence rates, reduced visual acuity and higher complication risks.

**Conclusion.** Biofilm formation by fungal pathogens significantly reduces antifungal susceptibility and worsens clinical outcomes in fungal keratitis. Elevated MIC, delayed healing and increased rates of complications emphasize the need for targeted biofilm-disrupting therapies and standardized diagnostic protocols. Future research should focus on developing clinical strategies that integrate biofilm assessment to improve patient outcomes.

## Data Summary

The data underlying this systematic review are available upon reasonable request. Extracted data from included studies (e.g. study characteristics, risk of bias assessments, outcome measures), data extraction forms and the full search strategy for each database are available from the corresponding author at annanurutami@mail.ugm.ac.id. No individual patient data were collected or analysed in this review. Statistical code used for the meta-analysis can also be shared upon request. All shared materials will be available for non-commercial use and will require appropriate citation. There are no restrictions on data use beyond those stated.

## Introduction

Corneal ulcer is a condition characterized by the loss of the epithelial layer of the cornea, often involving the underlying stroma, and can result in serious complications such as corneal scarring, perforation, posterior synechiae, glaucoma and cataract [1,4]. Infective corneal ulcers can be caused by micro-organisms such as bacteria, fungi, viruses or parasites [1,5, 6]. Fungal keratitis occurs most frequently in tropical and subtropical regions, with the highest incidence reported in Asia and Africa [7,14]. The most frequently identified fungal pathogens include *Aspergillus*, *Fusarium* and *Candida*. Fungal corneal ulcers tend to have worse clinical outcomes compared with bacterial ulcers [15,16]. Likewise, patients suffering from fungal ulcers have a higher likelihood of developing corneal perforations and needing keratoplasty than patients with bacterial ulcers [17,19].

Managing fungal keratitis is particularly difficult due to the complex behaviour of fungal pathogens, which can evade immune defences and develop resistance to antifungal treatments. One main factor is the formation of fungal biofilms, which play a major role in disease progression and therapeutic failure [19]. These biofilms are structured communities of fungal cells embedded in a protective extracellular matrix, shielding them from antifungal agents and harsh environmental conditions [20,22]. This matrix has two functions: providing structural support and augmenting survival against both drugs and the immune response. In fungal keratitis, biofilms can form on the corneal surface or within the ulcer bed, serving as reservoirs of infection that promote chronic disease and treatment resistance [23]. Biofilm-associated organisms are found to express extremely low metabolic activity while changing some of their gene expression, making them highly tolerant towards antifungal agents in contrast to those existing in suspension, also known as planktonic [24].

Unlike bacterial treatments, with multiple antibiotic options, antifungal agents are limited and predominantly fungistatic. This makes fungal ulcers harder to treat. Fungi counteract antifungal drugs through several mechanisms, such as upregulation of efflux pumps, modification of drug targets, increased enzyme activity of drug degradation and enhanced biofilm resilience [25]. Biofilm-associated infections are often refractory to standard antifungal regimens. As a result, patients experience prolonged healing times, higher recurrence rates and an increased risk of complications, such as corneal perforation and vision loss. Moreover, biofilms can impede drug penetration and promote the emergence of resistant fungal strains, complicating therapeutic strategies.

Despite growing recognition of the role of biofilms in fungal keratitis, there are still limited studies synthesizing the extent to which biofilm formation influences antifungal susceptibility and clinical outcomes. Variability in biofilm detection methods, heterogeneity in fungal species studied and differences in clinical settings have limited the ability to draw definitive conclusions. This systematic review and meta-analysis seek to fill existing knowledge gaps by compiling and analysing current evidence on how biofilm formation influences treatment outcomes in fungal corneal ulcers. The study explores microbiological, clinical and therapeutic aspects, emphasizing how biofilm formation affects treatment outcomes, healing duration and antifungal susceptibility.

## Methods

### Search strategy

This study complied with the PRISMA (Preferred Reporting Items for Systematic reviews and Meta-Analyses) 2020 guidelines and was registered under PROSPERO (an international systematic review registry, ID: CRD420251017502), with an available protocol (available in the online Supplementary Materials). The literature search was conducted in April 2025 on four databases, namely PubMed, ScienceDirect, the Cochrane Library and Scopus. The search terms used were [(corneal ulcer) OR (keratitis)] AND (fungal) AND (biofilm). This study included randomized controlled trials (RCTs) and observational studies. Exclusion criteria were non-English language articles, reviews, case reports, conference abstracts without full data and studies lacking clear biofilm assessment or outcome reporting.

Two independent investigators conducted the title and abstract screening. Another investigator supervised the screening process. The eligibility assessments of all articles were performed by both investigators independently. The investigators were not blinded to the sources’ bibliographic data.

### Eligibility criteria

Studies were selected for review according to the following inclusion criteria: (1) populations with fungal corneal ulcers or keratitis, (2) presence of biofilm-forming pathogens and (3) studies with clinical trials, RCTs, observational studies and *in vitro* studies. This review article excluded: (1) studies in patients with non-infectious corneal ulcers or other corneal pathologies, (2) studies without clear methodology for biofilm assessment and (3) animal studies.

### Data extraction and quality assessment

Data were extracted, which included clinical outcome measures, fungal species isolated, patient demographics, biofilm detection techniques (e.g. confocal microscopy, crystal violet staining and molecular assays), antifungal susceptibility testing protocols (including antifungal agents tested, MIC determination methods and interpretive criteria) and detailed study characteristics (author, year, country and study design). *In vitro* and clinical studies were analysed and reported separately to avoid inappropriate data pooling. Additional data on treatment regimens, follow-up duration and study quality indicators were also collected when available. Attempts were made to contact corresponding authors for clarification or missing data where applicable, but no replies were obtained.

Quantitative synthesis of MIC values was performed using random-effects meta-analyses with the Restricted Maximum Likelihood (REML) estimator to account for between-study variance. Between-study heterogeneity was quantified using τ², τ and I² statistics, and significance was assessed with Cochran’s Q test. Subgroup analyses were pre-specified by antifungal agent (voriconazole, amphotericin B, natamycin) to explore heterogeneity sources. The meta-analysis was conducted using the established statistical software RStudio 2025.05.0, with forest plots generated to visually represent MIC distributions and confidence intervals (CIs). Funnel plots were created to test publication bias in the studies. A narrative synthesis was performed for clinical outcomes, such as healing time, recurrence, visual acuity and complications.

All studies included were assessed using appropriate tools tailored to each study design for their risk of bias: ROBINS-I (Risk of Bias in Non-randomized Studies of Interventions) for clinical studies and the QUIN tool (Quality *In Vitro* Research tool) for *in vitro* experimental studies. Two independent investigators performed the risk of bias assessment. Another investigator resolved any disagreements that occurred.

## Results

### Flow of the study

**Fig. 1.**
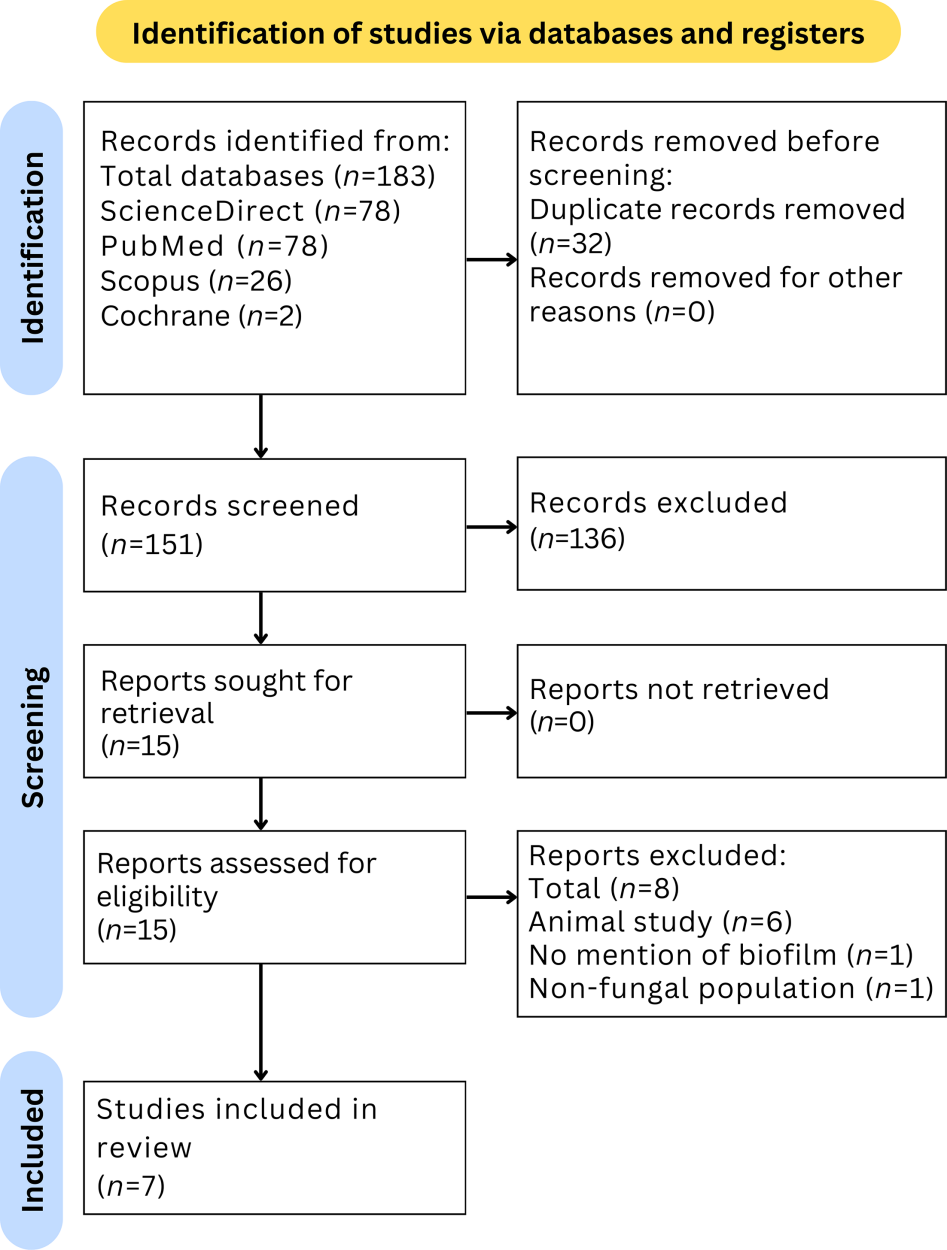
PRISMA diagram.

Fig. 1 describes the flow of this study using PRISMA protocols. Of the 183 articles, 15 were assessed for eligibility. Eight articles were excluded because they were animal studies (*n*=6), did not mention biofilm formation (*n*=1) or were in non-fungal populations (*n*=1). Hence, a total of seven articles were included in the systematic review for the final analysis.

### Study characteristics

Seven studies were included, with a combination of *in vitro* experimental designs, cross-sectional studies, observational clinical studies and hybrid formats. The study characteristics can be seen in Table 1. These studies were conducted in four countries: Brazil (four studies), China, Mexico and India. Three of the included studies were *in vitro* laboratory experiments using fungal isolates derived from patients with keratitis, without direct clinical follow-up [26,28]. These studies focused on biofilm formation and antifungal susceptibility. One study additionally performed proteomic analysis. The remaining four studies [29,32] integrated laboratory assays with clinical data, assessing outcomes such as treatment response, visual acuity and disease severity. One study also performed the phenotypic analysis of virulence factors.

**Table 1.** Study characteristics (*n*=7)

Author (year)	Country	Study type	Participant	Method of assessment	Primary outcome	Secondary outcome
Zhang *et al.* (2012) [26]	China	*In vitro* study	Three isolates from fungal keratitis patients (*Fusarium solani*, *Cladosporium sphaerospermum* and *Acremonium implicatum*)	Biofilm architecture: scanning electron microscopy and CSLM Antifungal susceptibility: CLSI M38-A method and XTT assay	Biofilm formation and architecture	Antifungal susceptibility
Calvillo-Medina *et al.* (2019) [27]	Mexico	*In vitro* study	One isolate from keratitis infectious agent (*Fusarium falciforme*)	Biofilm formation and architecture: colourimetric methods and by optical and electron microscopy Proteome assay: biofilm and planktonic cultures Proteomic analysis: 2-DE, protein identification and database search, real-time/reverse transcription PCR analysis	Biofilm formation and architecture	Protein expression
de Chaves *et al.* (2020) [28]	Brazil	*In vitro* experimental study	Ten isolates from patients with fungal keratitis (*Fusarium* sp.)	Biofilm formation: crystal violet Antifungal susceptibility: CLSI for filamentous fungi M38-A2 broth microdilution method, MTT colorimetric reagent Cytotoxicity assay: cell culture, cell viability assay, toxicity prediction by computational analysis	Biofilm formation	Antifungal susceptibility Biofilm removal and time–kill assays Cytotoxicity assay
Ranjith *et al.* (2017) [29]	India	Cross-sectional and *in vitro* study	Fifty isolates and patients with keratitis, endophthalmitis and orbital cellulitis (*Candida* sp. and non-*Candida* sp.)	Isolate identification: Vitek 2 compact system, DNA sequencing of ITS1-5.8S-ITS2 regions of the rRNA gene, phenotypic analysis of virulence factors Biofilm formation: microtitre 96-well TCP method Antifungal susceptibility: CLSI M27-S4 microbroth dilution method or agar diffusion E test	Antifungal susceptibility Biofilm formation	Phenotypic characterization of virulence factors Clinical outcomes including visual acuity
Bezerra *et al.* (2023) [30]	Brazil	Prospective observational and *in vitro* study	Thirteen isolates and patients with keratitis (*Candida* sp.)	Isolate identification: micromorphology analysis using chromogenic medium CHROMagar^TM^ Biofilm formation: crystal violet assay Biofilm metabolic activity: XTT reduction assay Antifungal susceptibility: CLSI M60 2017 broth microdilution method	Clinical outcomes including visual acuity	Biofilm formation Antifungal susceptibility
da Cunha Neto *et al.* (2024) [31]	Brazil	Prospective observational and *in vitro* study	Fifty isolates and patients with suspected keratitis (*Neocosmospora* sp. and *Fusarium* sp.)	Isolate identification: DNA extraction, PCR assay and DNA sequencing, phylogenetic and morphological analysis Biofilm formation: crystal violet assay Antifungal susceptibility: broth microdilution method	Clinical outcomes and epidemiological data	Molecular identification and phylogenetic analysis Biofilm formation Antifungal susceptibility
de Souza *et al.* (2025) [32]	Brazil	Cross-sectional and *in vitro* study	Fourteen isolates and patients with keratitis (FSSC and non-FSSC)	Isolate identification: phylogenetic analysis based on partial sequences of the RPB2 and TEF1α genes Biofilm formation and architecture: crystal violet assay, confocal microscopy Antifungal susceptibility: CLSI M38 microdilution protocol	Antifungal susceptibility	Biofilm formation Biofilm morphological analysis Clinical outcomes

CLSI, Clinical and Laboratory Standards Institute; CSLM, confocal scanning laser microscopy; 2-DE, two-dimensional gel electrophoresis; FSSC, * Fusarium solani* species complex; MTT, 3-(4,5-dimethylthiazol-2-yl)-2,5-diphenyltetrazolium bromide; RPB2, RNA Polymerase II subunit B; TCP, tissue culture plate; TEF1α, translation elongation factor 1-alpha; XTT, 2,3-bis-(2-methoxy-4-nitro-5-sulfophenyl)-2H-tetrazolium-5-carboxanilide.

Across all studies, biofilm formation and antifungal susceptibility were the most consistently reported primary outcomes, while secondary outcomes included clinical response, such as visual acuity, recovery and disease severity.

### Risk of bias assessment

**Fig. 2.**
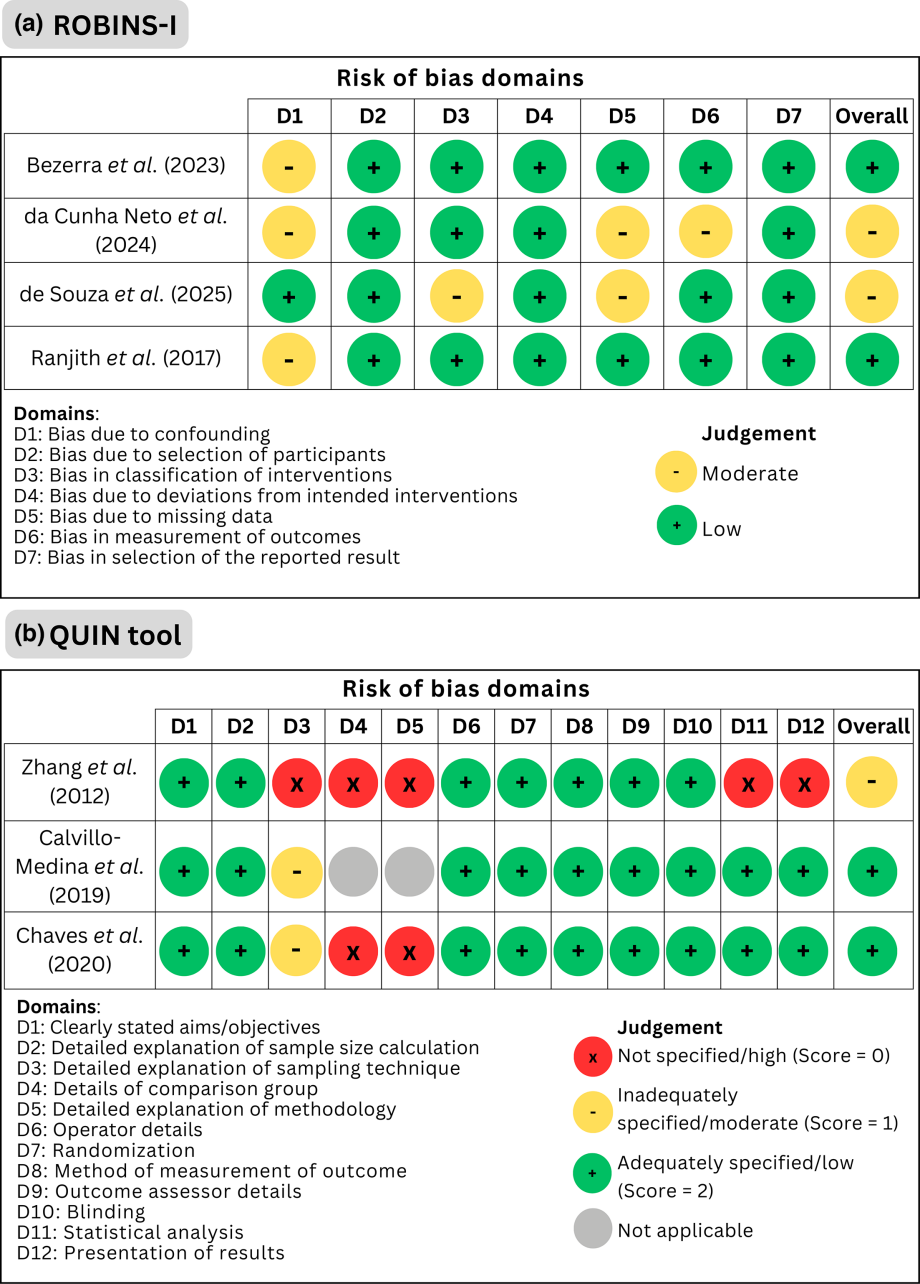
Risk of bias assessment. (a) ROBINS-I risk of bias assessment, and (b) QUIN tool risk of bias assessment.

The results of the risk of bias assessment are shown in Fig. 2. The risk of bias assessments for the included studies were conducted using ROBINS-I and the QUIN tool. Among the four studies evaluated using the ROBINS-I tool, two were evaluated as having an overall low risk of bias, while the remaining two studies were considered to have a moderate risk of bias (Fig. 2a).

The *In vitro* studies’ risk of bias assessment is shown in Fig. 2b. Based on this framework, Zhang *et al.* [26] received a score of 58, correlating to a moderate risk of bias. Calvillo-Medina *et al.* [27] achieved a score of 85, and de Chaves *et al.* [28] scored 75, both of which were classified as having a low risk of bias.

### Meta-analysis on antifungal susceptibility (*in vitro* studies)

The meta-analysis of antifungal susceptibility in biofilm-forming fungal isolates from keratitis cases synthesized data from three studies: Zhang *et al.* [26], de Chaves *et al.* [28], and Bezerra *et al.* [30]. A total of 22 fungal keratitis isolates were included across these studies. Antifungal susceptibility testing was performed for three antifungal agents: voriconazole (22 isolates), natamycin (13 isolates) and amphotericin B (12 isolates). These studies examined fungal species, predominantly *Fusarium* and *Candida*, isolated from clinical cases of fungal keratitis, and assessed their susceptibility to commonly used antifungal agents in the context of biofilm formation. The primary outcome measure was the MIC, which quantifies the lowest concentration of an antifungal agent required to inhibit fungal growth. Elevated MIC values in biofilm-associated isolates indicate increased resistance, posing significant challenges for effective treatment.

**Fig. 3.**
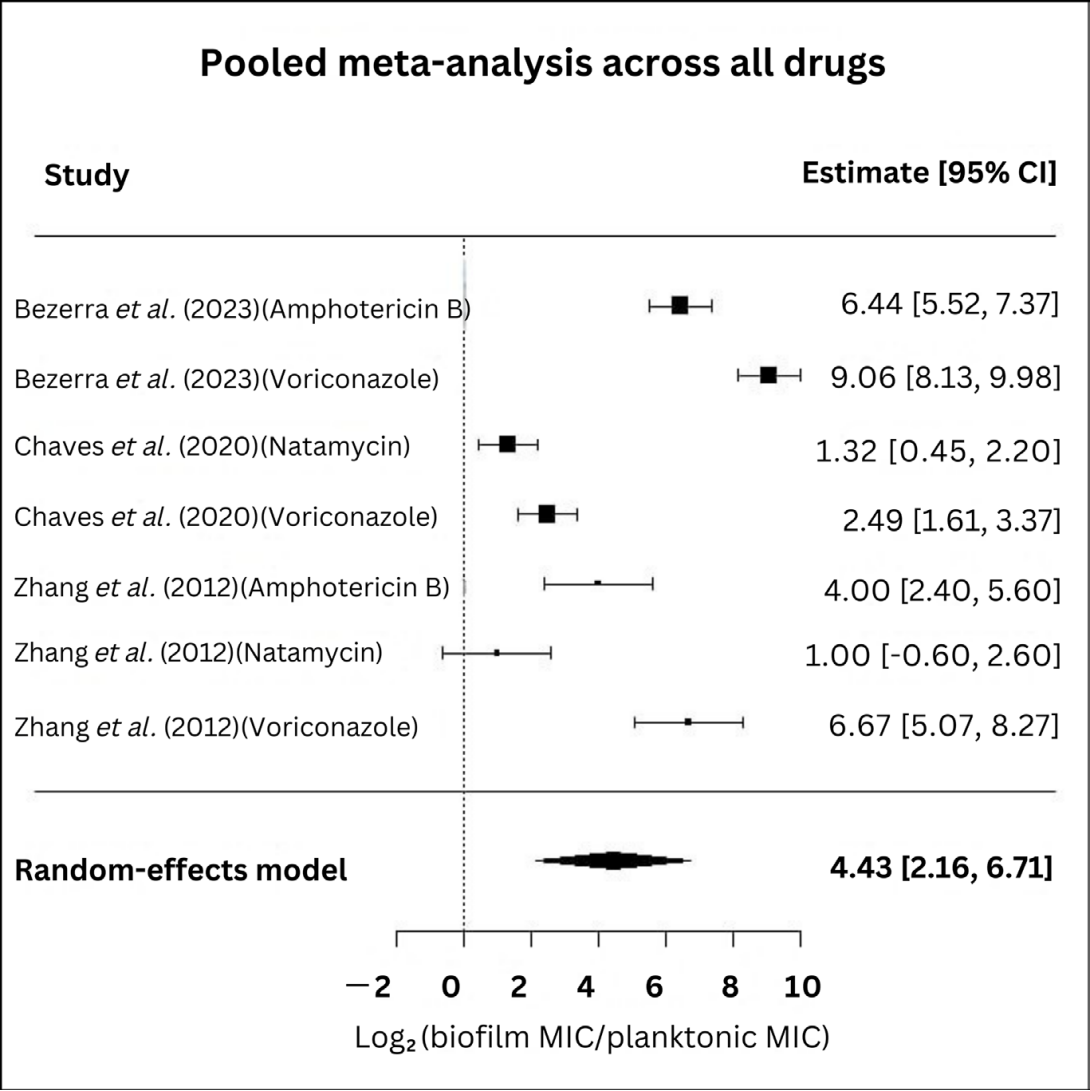
Pooled forest plot of antifungal efficacy against fungal biofilms compared with planktonic cells.

The pooled analysis of all drugs comparing planktonic cells with biofilm cells is shown in Fig. 3. Meta-analysis of the log₂-transformed ratio of biofilm MIC to planktonic MIC for antifungal treatment demonstrated significantly higher MIC values for biofilm-forming fungi compared with their planktonic counterparts. The pooled effect size across studies was 4.43 (95% CI: 2.16–6.71), indicating reduced susceptibility in biofilm states, reinforcing the finding that biofilm-associated fungal cells require significantly higher antifungal concentrations than planktonic cells. Heterogeneity was high (τ²=9.0225; I²=96.68%), with a significant heterogeneity test (Q=207.05; *P*≤0.0001). This suggests a consistent trend of biofilm-associated resistance across studies, despite heterogeneity in effect sizes. According to GRADE (Grading of Recommendations Assessment, Development, and Evaluation), the certainty of evidence for increased antifungal MIC in biofilm-forming isolates was very low, due to high heterogeneity (I²>85%), the small number of studies and the indirectness of *in vitro* evidence.

**Fig. 4.**
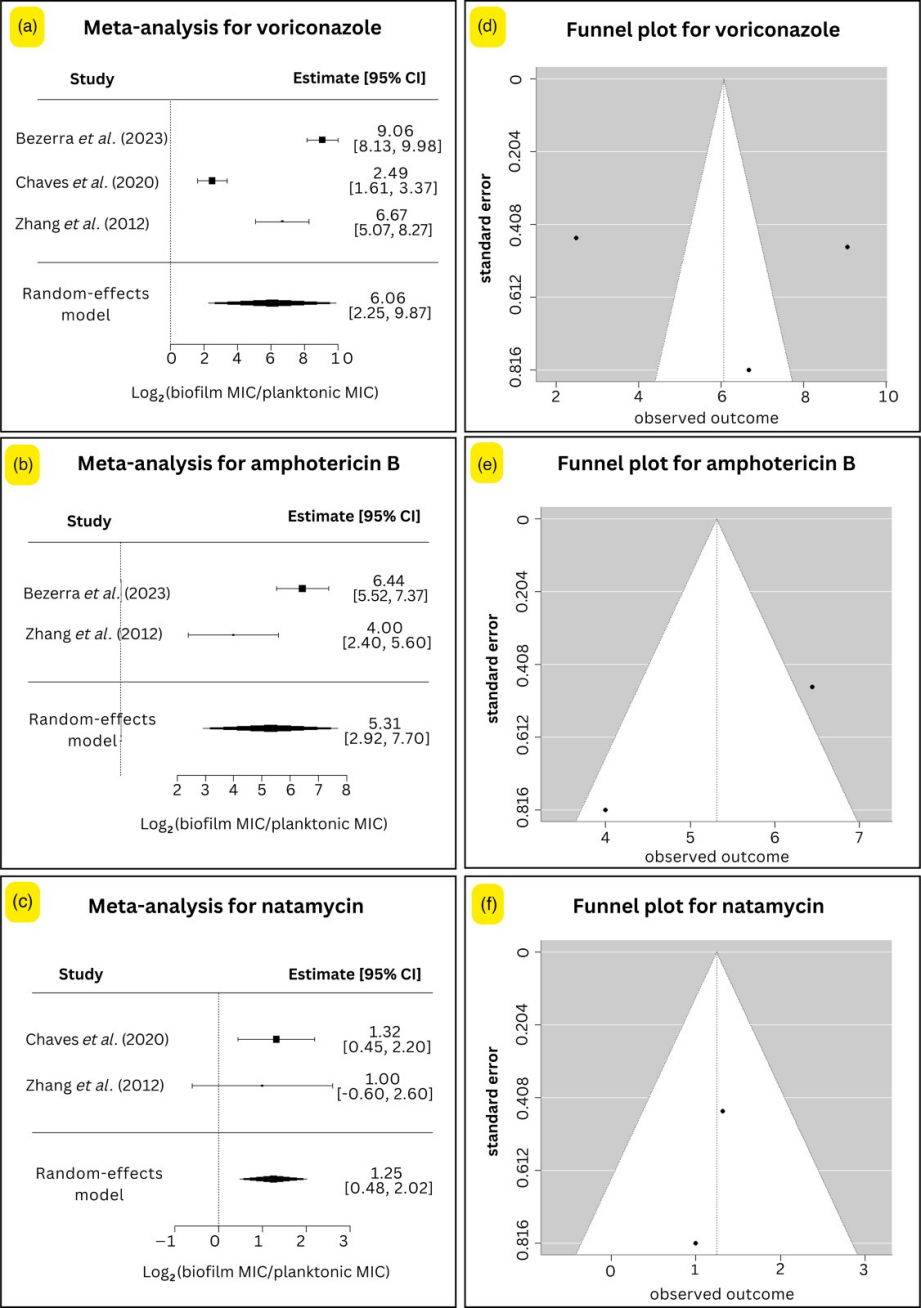
Subgroup forest plot and funnel plot bias assessment of antifungal efficacy against fungal biofilms compared with planktonic cells. (a–c) Forest plots representing the log_2_-transformed fold changes in MIC for biofilm versus planktonic forms of fungi when treated with (**a**) voriconazole, (**b**) amphotericin B and (**c**) natamycin. (d–f) Funnel plots for publication bias evaluation for (**d**) voriconazole, (**e**) amphotericin B and (**f**) natamycin.

Subgroup analysis of three commonly used drugs (voriconazole, amphotericin B and natamycin) was also performed (Fig. 4). The analysis revealed varying degrees of susceptibility reduction associated with biofilm-forming fungi, with differences in effect sizes and heterogeneity across antifungals.

#### Voriconazole

Three studies (*n*=22 isolates) contributed to the analysis of voriconazole, yielding a pooled effect size of 6.06 (95% CI: 2.25–9.87). This reflects a substantial reduction in voriconazole susceptibility in biofilm-forming fungi (*P*=0.0018). The heterogeneity was very high (τ²=10.9846; I²=97.4%), and the Q-test was significant (Q=103.71; *P*<0.0001), highlighting inconsistencies across the included studies.

#### Amphotericin B

The meta-analysis included two studies (*n*=12 isolates) examining the effect of amphotericin B. The pooled effect size was 5.31 (95% CI: 2.92–7.70), indicating a statistically significant increase in MIC in biofilm-associated fungal isolates (*P*<0.0001). Heterogeneity was high (τ²=2.5432; I²=85.1%). The significant heterogeneity test (Q=6.72; *P*=0.0095) supports the presence of differences in study populations or methodologies.

#### Natamycin

Two studies (*n*=13 isolates) assessed natamycin, producing a more modest but still significant pooled effect size of 1.25 (95% CI: 0.48–2.02; *P*=0.0015). Importantly, there was no observed heterogeneity (τ²=0; I²=0%), and the Q-test was non-significant (Q=0.12; *P*=0.73), indicating consistent results between studies. This suggests that natamycin’s antifungal activity is less affected by biofilm formation compared with other agents analysed.

### Clinical outcomes (clinical studies)

Clinical outcomes associated with biofilm formation in fungal keratitis, such as healing time, recurrence rates, visual acuity and complications, were examined through a narrative synthesis due to the limited and heterogeneous data available.

Healing time was reported in only one study. The data suggested a trend towards prolonged healing in cases involving biofilm-forming fungal isolates. It was noted that biofilm presence correlated with delayed corneal ulcer closure and extended antifungal treatment duration. This delay is likely attributable to the protective nature of biofilms, which shield fungal cells from antifungal agents and host immune responses, thereby sustaining infection and inflammation.

Recurrence rates were similarly reported in four studies and were often defined variably, including clinical deterioration or the need for additional antifungal therapy after initial resolution. This indicated that biofilm formation may contribute to higher recurrence rates, as biofilms can harbour dormant fungal cells that evade eradication and later reinitiate infection.

Visual acuity outcomes were reported in three of the clinical studies, but the methods of assessment and reporting scales varied, ranging from Snellen chart measurements to qualitative descriptions of vision improvement or deterioration. The available data suggested that biofilm formation was associated with poorer visual outcomes, likely due to prolonged infection, increased corneal scarring and complications such as stromal melting.

Complications, including corneal perforation, secondary bacterial infections and the need for surgical interventions like therapeutic keratoplasty, were reported in four studies. Biofilm-forming fungal infections frequently had more complications, reflecting the aggressive and refractory nature of biofilm-associated keratitis. Prolonged healing times, increased recurrence, poorer visual prognosis and higher complication rates emphasize the clinical significance of biofilms. According to GRADE, the certainty of evidence for all clinical outcomes was very low due to the observational design, heterogeneity and small sample sizes.

## Discussion

Biofilm formation in fungal keratitis has a significant clinical burden, so to maximize patient care, a thorough grasp of its implications is required. Biofilm formation leads to increased antifungal resistance, as shown by consistently high MIC values in isolates associated with biofilms in numerous studies [33]. As seen from the meta-analysis, three studies show that, despite the antifungal drug, the presence of biofilms drastically increases the MIC. However, it is important to note that, due to the small number of studies, statistical power and generalizability are limited.

Biofilms are advanced, complex structures that consist of fungal cells within an ECM (extracellular matrix) produced by the fungus itself [19,34,36]. Several mechanisms exist to confer resistant characteristics towards antifungals [37,38]. The ECM protective matrix, also referred to as the cover, consists of DNA, proteins, lipids and polysaccharides [25]. This hinders access to the target fungal cells by forming a physical barrier to antifungal diffusion or penetration [39]. In the subgroup analysis, we found that natamycin’s antifungal activity is less affected by biofilm formation compared with other agents analysed. This finding is similar to the Mycotic Ulcer Treatment Trial 1, in which natamycin treatment was associated with significantly better clinical and microbiological outcomes than voriconazole treatment for filamentous fungal keratitis [40,42].

In addition to enhanced membrane protection, biofilm cells are known to shift their metabolic states [24,43]. Lowered metabolism causes biofilm-forming fungi to enter a dormant or lesser active state, decreasing the efficacy of antifungal agents designed to target metabolically active cells. These observations stem from metabolically distinct ensembles showing different proteomic profiles owing to the overriding control of abundant, metabolically active enzymes. These changes enhance survival under harsher conditions, such as oxidative stress or nutrient starvation, while reducing vulnerability to therapeutic agents [39,44]. Elevated biofilm cell expression of genes responsible for stress response regulators, cell wall synthesis and drug efflux pumps contributes to this.

In studies on *Fusarium* and *Candida* species, biofilm-forming isolates have been seen to have heightened expression of the CDR1 (Candida Drug Resistance 1) and MDR1 (Multidrug Resistance Protein 1) resistance genes, which is associated with higher MIC values and increased treatment challenges [45]. This difference comes from the biofilm environment, leading to a more complex signalling network response. Furthermore, the antifungal resistance of biofilms is further complicated by polymicrobial interactions [46]. Bacteria frequently coexist with fungi in mixed-species biofilms, resulting in fungal biofilms that are often more resistant to treatment [47,48]. These bacterial and fungal biofilms highlight the necessity to address polymicrobial therapeutic approaches for many micro-organism interventions, complicating treatment plans even further [49].

Clinical research by Bezerra *et al.* [30] and da Cunha Neto *et al.* [31] has demonstrated that biofilm-associated keratitis causes longer healing periods. These cases require careful management and long-lasting antifungal therapy. Infections that contain biofilm are also more persistent and likely to recur, requiring more extensive treatment or even surgery. Recovery of visual acuity is also impacted. Patients with biofilm-positive keratitis experience exacerbated symptoms due to chronic inflammation, plus corneal injury.

Novel therapeutic pathways are being explored to solve these problems. Some combination treatments, such as those based on clioquinol, have shown improvement due to enhanced drug delivery and reduced MIC within biofilms [28]. Early surgical intervention remains a viable option, especially in refractory cases. When medical therapy fails, early keratotomy or keratoplasty may become essential to excise the infected tissue and biofilm-covered structures [50].

In summary, treatment of biofilm-associated fungal keratitis requires a multifaceted approach, including proactive and responsive measures, such as early detection, thorough intervention, monitoring, tailored antifungal treatment, possible additional treatment and set procedures with comprehensive guidelines. Recognizing biofilm formation as a critical determinant of prognosis can inform better therapeutic planning and support improved outcomes for patients affected by this complex ocular infection.

## Limitations

The limitation of this systematic review is the pronounced heterogeneity in study designs. Differences in patient populations, fungal species investigated, geographic settings and clinical management protocols are thoroughly seen. This is due to the minimal number of studies regarding biofilms in corneal ulcers, leading to high heterogeneity. Although variability in assessment methods across studies complicates meta-analysis, the overall trend indicates that biofilm formation is associated with diminished healing and increased MIC. However, the clinical use of these data is limited. Risk of publication bias assessment using funnel plots is also limited in reliability due to the small number of studies per drug category.

Clinical outcomes such as healing time, visual acuity and recurrence were inconsistently reported across studies and were heterogeneous and did not allow meta-analysis. Reliance on narrative summaries is inherently less precise and more susceptible to interpretive bias.

Future research should develop standard methods for detecting biofilms and testing antifungal resistance in eye infections. More standardized research is also needed to better evaluate the effects of biofilm, mainly in difference in strength, on clinical outcomes. New clinical trials are needed to test antifungal drugs and combinations against biofilm-forming fungi. These trials should use uniform measures and follow patients long-term. Using molecular tests with clinical data can help guide treatment. Studies on how fungi in biofilms evade the immune system may reveal new targets for therapy.

## Conclusion

Biofilm formation by fungal pathogens in corneal ulcers significantly exacerbates antifungal resistance and adversely affects clinical outcomes. The elevated MIC values observed in biofilm-associated fungal isolates prove the barrier biofilms pose to effective antifungal therapy, often rendering standard treatments insufficient. This resistance is multifactorial, involving complex extracellular matrix production, altered fungal metabolism and gene expression changes.

Clinically, biofilm presence correlates with prolonged healing times, increased recurrence rates, poorer visual acuity and a higher incidence of complications such as corneal perforation and secondary infections. These outcomes not only prolong patient morbidity but also increase the likelihood of surgical interventions, thereby elevating healthcare costs and burden.

## Supplementary material

10.1099/jmm.0.002106 Uncited Supplementary Material 1.

## Abbreviations

CI: confidence interval
QUIN tool: quality *in vitro* research tool
RCT: randomized controlled trial
REML: restricted maximum likelihood
ROBINS-I: risk of bias in non-randomized studies of interventions
