# Supplementary material for: Impact of biofilm formation in fungal corneal ulcers on treatment outcomes: a systematic review and meta-analysis
Source: J Med Microbiol. 2025 Dec 18;74(12):002106. doi: 10.1099/jmm.0.002106 (PMC12738880; doi:10.1099/jmm.0.002106)
Supplement: Uncited Supplementary Material 1. [file jmm-74-02106-s001.pdf]

PRISMA 2020 Checklist

Section and Topic	Item #	Checklist item	Location where item is reported
**TITLE**			
Title	1	Identify the report as a systematic review.	Title
**ABSTRACT**			
Abstract	2	See the PRISMA 2020 for Abstracts checklist.	Abstract
**INTRODUCTION**			
Rationale	3	Describe the rationale for the review in the context of existing knowledge.	1
Objectives	4	Provide an explicit statement of the objective(s) or question(s) the review addresses.	1
**METHODS**			
Eligibility criteria	5	Specify the inclusion and exclusion criteria for the review and how studies were grouped for the syntheses.	2.2, 2.3
Information sources	6	Specify all databases, registers, websites, organisations, reference lists and other sources searched or consulted to identify studies. Specify the date when each source was last searched or consulted.	2.1
Search strategy	7	Present the full search strategies for all databases, registers and websites, including any filters and limits used.	2.1
Selection process	8	Specify the methods used to decide whether a study met the inclusion criteria of the review, including how many reviewers screened each record and each report retrieved, whether they worked independently, and if applicable, details of automation tools used in the process.	2.1, 2.2
Data collection process	9	Specify the methods used to collect data from reports, including how many reviewers collected data from each report, whether they worked independently, any processes for obtaining or confirming data from study investigators, and if applicable, details of automation tools used in the process.	2.1
Data items	10a	List and define all outcomes for which data were sought. Specify whether all results that were compatible with each outcome domain in each study were sought (e.g. for all measures, time points, analyses), and if not, the methods used to decide which results to collect.	2.3
	10b	List and define all other variables for which data were sought (e.g. participant and intervention characteristics, funding sources). Describe any assumptions made about any missing or unclear information.	2.3
Study risk of bias assessment	11	Specify the methods used to assess risk of bias in the included studies, including details of the tool(s) used, how many reviewers assessed each study and whether they worked independently, and if applicable, details of automation tools used in the process.	2.3
Effect measures	12	Specify for each outcome the effect measure(s) (e.g. risk ratio, mean difference) used in the synthesis or presentation of results.	2.3
Synthesis methods	13a	Describe the processes used to decide which studies were eligible for each synthesis (e.g. tabulating the study intervention characteristics and comparing against the planned groups for each synthesis (item #5)).	2.3
	13b	Describe any methods required to prepare the data for presentation or synthesis, such as handling of missing summary statistics, or data conversions.	2.3
	13c	Describe any methods used to tabulate or visually display results of individual studies and syntheses.	2.3
	13d	Describe any methods used to synthesize results and provide a rationale for the choice(s). If meta-analysis was performed, describe the model(s), method(s) to identify the presence and extent of statistical heterogeneity, and software package(s) used.	2.3
	13e	Describe any methods used to explore possible causes of heterogeneity among study results (e.g. subgroup analysis, meta-regression).	2.3
	13f	Describe any sensitivity analyses conducted to assess robustness of the synthesized results.	2.3
Reporting bias assessment	14	Describe any methods used to assess risk of bias due to missing results in a synthesis (arising from reporting biases).	3.3
Certainty assessment	15	Describe any methods used to assess certainty (or confidence) in the body of evidence for an outcome.	3.3

PRISMA 2020 Checklist

Section and Topic	Item #	Checklist item	Location where item is reported
**RESULTS**			
Study selection	16a	Describe the results of the search and selection process, from the number of records identified in the search to the number of studies included in the review, ideally using a flow diagram.	3.1
	16b	Cite studies that might appear to meet the inclusion criteria, but which were excluded, and explain why they were excluded.	Figure 1
Study characteristics	17	Cite each included study and present its characteristics.	Table 1
Risk of bias in studies	18	Present assessments of risk of bias for each included study.	3.3
Results of individual studies	19	For all outcomes, present, for each study: (a) summary statistics for each group (where appropriate) and (b) an effect estimate and its precision (e.g. confidence/credible interval), ideally using structured tables or plots.	3.4
Results of syntheses	20a	For each synthesis, briefly summarise the characteristics and risk of bias among contributing studies.	3.4
	20b	Present results of all statistical syntheses conducted. If meta-analysis was done, present for each the summary estimate and its precision (e.g. confidence/credible interval) and measures of statistical heterogeneity. If comparing groups, describe the direction of the effect.	3.4
	20c	Present results of all investigations of possible causes of heterogeneity among study results.	3.4
	20d	Present results of all sensitivity analyses conducted to assess the robustness of the synthesized results.	3.4
Reporting biases	21	Present assessments of risk of bias due to missing results (arising from reporting biases) for each synthesis assessed.	3.3
Certainty of evidence	22	Present assessments of certainty (or confidence) in the body of evidence for each outcome assessed.	3.3
**DISCUSSION**			
Discussion	23a	Provide a general interpretation of the results in the context of other evidence.	4
	23b	Discuss any limitations of the evidence included in the review.	4.1
	23c	Discuss any limitations of the review processes used.	4.1
	23d	Discuss implications of the results for practice, policy, and future research.	4.1
**OTHER INFORMATION**			
Registration and protocol	24a	Provide registration information for the review, including register name and registration number, or state that the review was not registered.	2.1
	24b	Indicate where the review protocol can be accessed, or state that a protocol was not prepared.	2.1
	24c	Describe and explain any amendments to information provided at registration or in the protocol.	No amandments
Support	25	Describe sources of financial or non-financial support for the review, and the role of the funders or sponsors in the review.	6
Competing interests	26	Declare any competing interests of review authors.	6
Availability of data, code and other materials	27	Report which of the following are publicly available and where they can be found: template data collection forms; data extracted from included studies; data used for all analyses; analytic code; any other materials used in the review.	7

From: Page MJ, McKenzie JE, Bossuyt PM, Boutron I, Hoffmann TC, Mulrow CD, et al. The PRISMA 2020 statement: an updated guideline for reporting systematic reviews. BMJ 2021;372:n71. doi: 10.1136/bmj.n71

SYSTEMATIC REVIEW PROTOCOL

**Title of the review**	**Impact of Biofilm Formation in Fungal Corneal Ulcers on Treatment Outcomes: A Systematic Review**
**Registration**	**PROSPERO (CRD420251017502)**
**Team reviewer**	Anna Nur Utami, M.D.<br><br>Alya Nabilah Tasya, M.D.
**Supervisors**	Prof. Tri Wibawa, M.D., Ph.D.<br><br>Rina La Distia Nora, M.D., Ph.D.
**Affiliations**	Universitas Gadjah Mada<br>JEC Eye Clinic and Hospitals
**Support**	No financial interest

INTRODUCTION

**Rationale**	Corneal ulcer is a destructive condition of the corneal tissue that can lead to permanent vision impairment or even blindness. It is characterized by the loss of the corneal epithelial layer, often involving the underlying stroma, and has the potential to cause serious complications such as corneal scarring, perforation, glaucoma, cataracts, and posterior synechiae.<br><br>In developing countries, infectious causes, particularly bacterial and fungal infections, are the most common causes of corneal ulcer. Corneal ulcer caused by fungi is known as an infectious eye disease with a prolonged healing time. This delayed healing is influenced by several factors, one of which is the biofilm formed by microorganisms, including both bacteria and fungi. Unlike bacterial infections, which have a variety of antibiotic options with different mechanisms, available antifungal agents are limited to four main classes: azoles, polyenes, echinocandins, and flucytosine. However, most available antifungals are fungistatic at commonly used concentrations rather than fungicidal. Limited drug penetration, particularly within the biofilm matrix, is one of the primary reasons why fungal ulcer treatment takes longer compared to bacterial ulcers. This biofilm structure is believed to create a physical barrier against drug penetration, thereby reducing the effectiveness of both topical and systemic antifungal treatments.<br><br>Fungi possess complex molecular mechanisms to counteract antifungal agents, including drug structure manipulation, reduction of drug concentration through efflux pumps, modification of drug target sites, and increased intracellular enzyme activity. Biofilm plays a crucial role in influencing chronicity. During biofilm formation, efflux pump activity facilitates biofilm maturation, making it difficult for topical drugs to penetrate. In biofilm conditions, 140 efflux pump genes, including ABC transporter *MDR1, MDR2,* and *MDR4*, are upregulated. The increase in antimicrobial resistance is associated with the maximal expression of the *MDR4* gene in mature biofilms.<br><br>These biofilms consist of a complex extracellular matrix that protects fungal cells from antifungal agents and enables resistance to harsh environmental conditions. This increases the risk of persistent and invasive infections, often necessitating the removal of medical devices and infected tissue. Biofilms produced by yeast are more similar to those formed by bacteria. This differs from the biofilms formed by filamentous fungi, which have unique morphological characteristics due to the absence of binary fission and the dynamic growth patterns typically observed in the asexual stage of yeast.

	Therefore, an in-depth study of the role of biofilm in the therapeutic response of fungal corneal ulcers is highly necessary to elucidate the molecular mechanisms of biofilm formation and resistance development, which could ultimately pave the way for more effective antifungal therapies.
**Objective**	To systematically review and synthesize the impact of biofilm formation on the chronicity and treatment outcomes of fungal corneal ulcers, including its role in virulence factors, delayed wound healing and decreased antimicrobial susceptibility.
**Research question**	How does biofilm formation influence the chronicity and treatment outcomes in fungal corneal ulcers?
**Type of outcome measures**	We included studies that report the treatment outcomes including microbiological biofilm characteristic, the rate of corneal wound healing and antimicrobial susceptibility.
**Primary Outcomes**	Treatment outcomes including microbiological biofilm characteristic, the rate of corneal wound healing, and antimicrobial susceptibility.
**Secondary outcomes**	Microbiological profile<br>Visual outcomes<br>Complication rate<br>Need for surgical interventions

METHODS

**Inclusion criteria**	
**Participants (P)**	Patients with corneal ulcers (fungal)

**Intervention (I)**	Presence of biofilm-forming pathogens
**Comparison (C)**	Non-biofilm-forming pathogens
**Outcome (O)**	**Primary outcome:** Treatment outcomes including the rate of corneal wound healing, antimicrobial susceptibility and microbiological biofilm characteristic.<br><br>**Outcome of measure:**<br><br>● Corneal wound healing:<br><br>Number of days until complete epithelial healing of the corneal ulcer. Examines corneal opacity, neovascularization, and residual scarring by slit-lamp biomicroscope, and AS-OCT<br><br>● Antimicrobial susceptibility:<br><br>antimicrobial sensitivity test also known as susceptibility test<br><br>● Microbiological biofilm characteristics:<br><br>Biofilm Isolation and Culture; Biofilm Morphology Observation microscopy (lactophenol cotton blue and calcofluor white (CW); Spectrophotometric Analysis of Biofilm Biomass formed in 96-well plates; Scanning Electron Microscope (SEM) Analysis; and biofilm metabolic activity assay using XTT Reduction Test<br><br>**Secondary outcomes:**<br><br>Microbiological profile: Microbiological culture test<br><br>Visual outcomes: Visual acuity measurement by Snellen chart written in LogMAR notation and / or contrast sensitivity test<br><br>Complications: such as glaucoma, cataract and corneal perforations.<br><br>Need for surgical interventions: Operations performed such as antimicrobial intrastromal/ intracameral injections, amnion membrane transplant, penetrating keratoplasty, periosteal graft, evisceration and dermofat graft.

**Study design (S)**	Clinical Trial, Randomized Control Trials and Observational studies
**Setting**	English full-text publications
**Exclusion criteria (not included in the inclusion criteria)**	
	Case reports, reviews, systematic reviews, meta-analysis and opinion pieces.<br>Studies that focus on non-corneal infections.<br>Studies without clear methodology on biofilm assessment.<br>Animal studies.

**Search strategy**	
**Electronic databases**	Pubmed/MEDLINE<br>Cochrane Library<br>Scopus<br>Science Direct
**Other methods used for identifying relevant research**	Reference checking of retrieved full text papers<br>Epistemonikos

**Search strategy**	**Pubmed/MEDLINE**<br><br>Search date: 19/03/2025	"corneal ulcer"[Title/Abstract] OR "keratiti*"[Title/Abstract] OR "ulcerative keratitis"[Title/Abstract] OR "microbial keratitis"[Title/Abstract] OR "infectious keratitis"[Title/Abstract] OR "corneal ulcer"[MeSH Terms]<br>AND<br>(biofilm*[Title/Abstract]) OR (biofilm[MeSH Terms])<br>AND<br>((("randomized"[Title/Abstract] OR "placebo"[Title/Abstract] OR "drug therapy"[MeSH Subheading] OR "randomly"[Title/Abstract] OR "trial"[Title/Abstract] OR "groups"[Title/Abstract]) NOT ("animals"[MeSH Terms] NOT "humans"[MeSH Terms])) NOT ("randomized controlled trial"[Publication Type] OR "controlled clinical trial"[Publication Type])) OR (("Observational Studies as Topic"[MeSH] OR "Cohort Studies"[MeSH] OR "Case-Control Studies"[MeSH] OR "Cross-Sectional Studies"[MeSH]))
	**Cochrane Library**<br><br>Search date: 21/03/2025	#1 ("corneal ulcer"):ti,ab,kw OR (keratiti*):ti,ab,kw OR ("microbial keratitis"):ti,ab,kw OR ("infectious keratitis"):ti,ab,kw OR ("mycotic keratitis"):ti,ab,kw 1460<br><br>#2 MeSH descriptor: [Corneal Ulcer] explode all trees 197<br><br>#3 ("biofilm"):ti,ab,kw OR (biofilm*):ti,ab,kw 1963<br><br>#4 MeSH descriptor: [Biofilms] explode all trees 493<br><br>#5 #1 OR #2 1460<br><br>#6 #3 OR #4 1963

		#7 #5 AND #6 2
	**Scopus**<br><br>Search date: 21/03/2025	TITLE-ABS-KEY ("corneal ulcer" OR keratitis OR "infectious keratitis" OR "microbial keratitis") AND TITLE-ABS-KEY (biofilm*) AND TITLE-ABS-KEY (("randomized controlled trial" OR "randomised controlled trial" OR "RCT" OR "randomized trial" OR "randomised trial" OR "controlled trial" OR "clinical trial" OR "controlled clinical trial" OR "intervention study" OR "experimental study" OR "cohort study" OR "longitudinal study" OR "prospective study" OR "retrospective study" OR "case-control study" OR "matched case-control study" OR "cross-sectional study" OR "prevalence study" OR "survey study"))
	**Science Direct**<br><br>Search date: 21/03/2025	("corneal ulcer" OR keratitis OR "ulcerative keratitis" OR "microbial keratitis"OR "infectious keratitis") AND (biofilm OR biofilms)

**Methods of review**	
**Selection process**	Titles and abstract of retrieved papers will undergo screening for relevance by two reviewers. Papers that are deemed irrelevant will be excluded from the review, and the full text of remaining papers will be obtained. Excluded papers will be documented, along with the reasons for exclusion.<br><br>Two reviewers will independently evaluate the eligibility of studies based on predefined inclusion and exclusion criteria. Any disagreement will be resolved through discussion and consensus with the involvement of the third and fourth reviewer.
**Critical appraisal**	Randomized Control Trials and Clinical studies: Cochrane Risk of Bias Tool (RoB 2.0)<br><br>Non randomized Clinical studies: ROBINS-I Tool

	Observational studies (cohort, case-control, and cross-sectional studies): Newcastle-Ottawa Scale (NOS) or Joanna Briggs Institute (JBI) Critical Appraisal Tools<br><br>Two independent reviewers will assess bias, and disagreements will be resolved by a third and fourth reviewer. Studies with a high risk of bias (NOS ≤4) will be excluded from systematic review. A sensitivity analysis will be conducted to determine the impact of study quality on overall results.
**Data extraction**	Following article appraisal, relevant data will be extracted for further review by two reviewers independently. Any disagreement will be resolved through discussion and consensus with the involvement of the other reviewers.<br><br>Information extracted from the studies includes:<br><br>· Study characteristics (author, year, country, study design)<br><br>· Population (type of sample, sample size)<br><br>· Intervention details (Biofilm producing microorganisms)<br><br>· Outcomes (treatment outcomes including wound healing time, antimicrobial susceptibility and microbiological biofilm characteristics. Secondary outcomes including visual outcomes, microbiological profile, complications and need for surgical interventions)<br><br>· Follow-up duration
**Data synthesis**	Qualitative synthesis for all included studies<br><br>Meta-analysis (if sufficient homogeneous data are available)
**Meta-analysis**	The feasibility of a meta-analysis can only become apparent once all the data has been extracted within the reviewing process
**Ethics and Dissemination**	Ethical approval is not required for this review. The findings will be disseminated through peer-reviewed publications and conference presentations.

**References**	1. Sharma S, Singh S, Goel S, Farooq U. Role of biofilms in fungal keratitis: an overview. J Bacteriol Mycol Open Access. 2017;5(6):409–412. DOI: 10.15406/jbmoa.2017.05.00157.<br>2. Yi J, Sun Y, Zeng C, Kostoulias X, Qu Y. The Role of Biofilms in Contact Lens Associated Fungal Keratitis. Antibiotics (Basel). 2023 Oct 12;12(10):1533. doi: 10.3390/antibiotics12101533<br>3. Ali A, Zahra A, Kamthan M, Husain FM, Albalawi T, Zubair M, Alatawy R, Abid M, Noorani MS. Microbial Biofilms: Applications, Clinical Consequences, and Alternative Therapies. *Microorganisms*. 2023; 11(8):1934. https://doi.org/10.3390/microorganisms11081934<br>4. Gintjee TJ, Donneley MA, Thomson GR. Aspiring antifungals: review of current antifungal pipeline development. 2020. JoF. 6(1):28.<br>5. Darsini IP. Demographic profile, clinical characteristics and therapy of keratitis and corneal ulcers in the Ophthalmology Clinic of Infection and Immunology Kirana Cipto Mangunkusumo Hospital period of January – December; 2013. Descriptive Study. Jakarta: Universitas Indonesia; 2015.<br>6. Pascolini D, Mariotti SP. Global estimates of visual impairment: 2010. Br J Ophthalmol. 2012 May;96(5):614-8. doi: 10.1136/bjophthalmol-2011-300539. Epub 2011 Dec 1. PMID: 22133988.<br>7. Sharma N, Bagga B, Singhal D, et al. Fungal keratitis: A review of clinical presentations, treatment strategies and outcomes. *Ocul Surf*. 2022;24:22-30. doi:10.1016/j.jtos.2021.12.001<br>8. Urwin L, Okurowska K, Crowther G, Roy S, Garg P, Karunakaran E, MacNeil S, Partridge LJ, Green LR, Monk PN. Corneal Infection Models: Tools to Investigate the Role of Biofilms in Bacterial Keratitis. *Cells*. 2020; 9(11):2450. https://doi.org/10.3390/cells9112450<br>9. Yi J, Sun Y, Zeng C, Kostoulias X, Qu Y. The Role of Biofilms in Contact Lens Associated Fungal Keratitis. Antibiotics (Basel). 2023 Oct 12;12(10):1533. doi: 10.3390/antibiotics12101533<br>10. Costa-Orlandi CB, Sardi JCO, Pitangui NS, De Oliveira HC, Scorzoni L, Galeane MC, Medina-Alarcón KP, Melo WCMA, Marcelino MY, Braz JD, et al. Fungal Biofilms and Polymicrobial Diseases. *Journal of Fungi*. 2017; 3(2):22. https://doi.org/10.3390/jof3020022<br>11. Shivaji S, Nagapriya B, Ranjith K. Differential Susceptibility of Mixed Polymicrobial Biofilms Involving Ocular Coccoid Bacteria (*Staphylococcus aureus* and *S. epidermidis*) and a Filamentous Fungus (*Fusarium solani*) on Ex Vivo Human Corneas. *Microorganisms*. 2023; 11(2):413. https://doi.org/10.3390/microorganisms11020413<br>12. Calvillo-Medina RP, Martínez-Neria M, Mena-Portales J, et al. Identification and biofilm development by a new fungal keratitis aetiologic agent. *Mycoses*. 2019;62(1):62-72. doi:10.1111/myc.12849

	Inclusion Criteria	Exclusion Criteria
**P**<br>Participants	All patients with fungal corneal ulcer	- Patients with infectious non-fungal corneal ulcer<br>- Patient with non infectious corneal ulcer<br>- Patient with other corneal pathologies
**I**<br>Intervention	Presence of biofilm-forming pathogens	-
**C**<br>Control	Non-biofilm-forming pathogens	-
**O**<br>Outcome	The primary outcomes are wound healing time, antimicrobial susceptibility and microbiological biofilm characteristics.	-
**S**<br>Study Design	Original research papers, Clinical Trials, Randomized Control Trials, and Observational studies	-
**Language**	English	Other than english
**Setting**	All settings	None

TIMELINE FOR REVIEW

**Protocol**	2 weeks
**Literature searching**	1 month
**Data extraction**	2 weeks
**Quality assessment**	2 weeks
**Writing up**	2 months
